# Global Regulation of Genetically Modified Crops Amid the Gene Edited Crop Boom – A Review

**DOI:** 10.3389/fpls.2021.630396

**Published:** 2021-02-24

**Authors:** Crystal Turnbull, Morten Lillemo, Trine A. K. Hvoslef-Eide

**Affiliations:** Faculty of Biosciences, Institute of Plant Science, Norwegian University of Life Sciences (NMBU), Ås, Norway

**Keywords:** regulations (laws), gene edited crops, legislation, cultivating GMO crops, new breeding techniques, GMO crops, harmonization, biosafety

## Abstract

Products derived from agricultural biotechnology is fast becoming one of the biggest agricultural trade commodities globally, clothing us, feeding our livestock, and fueling our eco-friendly cars. This exponential growth occurs despite asynchronous regulatory schemes around the world, ranging from moratoriums and prohibitions on genetically modified (GM) organisms, to regulations that treat both conventional and biotech novel plant products under the same regulatory framework. Given the enormous surface area being cultivated, there is no longer a question of acceptance or outright need for biotech crop varieties. Recent recognition of the researchers for the development of a genome editing technique using CRISPR/Cas9 by the Nobel Prize committee is another step closer to developing and cultivating new varieties of agricultural crops. By employing precise, efficient, yet affordable genome editing techniques, new genome edited crops are entering country regulatory schemes for commercialization. Countries which currently dominate in cultivating and exporting GM crops are quickly recognizing different types of gene-edited products by comparing the products to conventionally bred varieties. This nuanced legislative development, first implemented in Argentina, and soon followed by many, shows considerable shifts in the landscape of agricultural biotechnology products. The evolution of the law on gene edited crops demonstrates that the law is not static and must adjust to the *mores* of society, informed by the experiences of 25 years of cultivation and regulation of GM crops. The crux of this review is a consolidation of the global legislative landscape on GM crops, as it stands, building on earlier works by specifically addressing how gene edited crops will fit into the existing frameworks. This work is the first of its kind to synthesize the applicable regulatory documents across the globe, with a focus on GM crop cultivation, and provides links to original legislation on GM and gene edited crops.

## Introduction

Agriculture in the 21st century feeds, clothes, and fuels billions of people, with fewer farmers, limited land availability and additional modern challenges. Yet, humans have always found a way to build on previous knowledge to improve agricultural capabilities and it is these improvements that have led to higher production and access. The employment of biotechnology is just one part of agricultural innovation that contributes to modern agricultural success. As with any new technology, risks associated therewith must be assessed and managed, a task that in the last 30 to 40 years has fallen to the legislators (Levin, 1994; Aven, 2016). Safety of the food we eat, feed for animals and protection of the environment remain central criteria to the risk assessment when employing agricultural technology. These criteria are applied to all agricultural products destined for consumption and use, including those varieties bred using traditional plant breeding methods. Determination of a new crop or plant product as a “genetically modified organism” or GMO is one method that has evolved to trigger national regulations, which regulations contain applicable risk assessments and management strategies. The assessment depends on whether the product is destined as food (regulatory framework for food), feed (regulatory framework for animal feed) or for cultivation purposes (regulatory framework for agriculture and/or the environment), the development of which comes with a host of associated challenges (Huesing et al., 2016).

In the last 25 years, GM crop production has experienced over 100-fold increase (Brookes and Barfoot, 2013; Mathur et al., 2017). Currently, farmers cultivate approximately 190 million hectares of biotech crops, which is approximately equivalent to the entire surface area of Mexico (ISAAA, 2020b). Soybean (∼50%), maize (∼30%), cotton (∼13%) and canola (∼5%) make up the four primary cultivated crops (ISAAA, 2018, 2020b). Most of the products of the four major crops are not traditionally destined for human consumption (Aldemita et al., 2015). Soybean crops provide soybean oil, which is a versatile component in vegetable oil products as well as industrial adhesives, solvents and lubricants whilst the bean meal is a high protein constituent in animal feed (Nosowitz, 2017). GM cotton accounts for 79% of total cotton cultivation and remains an important natural source of fiber (Townsend, 2019; ISAAA, 2020b). On the other hand, utilization of maize has shifted from animal feed to ethanol production in the last two decades, particularly in the United States (Wallington et al., 2012; Klopfenstein et al., 2013; Ranum et al., 2014). According to the Food and Agriculture Organization (FAO), of the total global production of maize, 55% was utilized as feed, 20% to other non-food uses and only 12% as food (FAO, 2020).

This review will expand on the articles discussing the regulatory status of GM crops, such as Nap et al. (2003) and Ishii and Araki (2017), and build on these works by specifically addressing how gene edited crops will fit into the existing frameworks. This work is the first of its kind to synthesize the applicable regulatory documents across the globe, with a focus on GM crop cultivation, and provides links to original legislation on GM and gene edited crops. Certain countries have been addressed extensively in the literature on their unique legislation, particularly in the western countries (the European Union, the United States, Canada, Australia, etc.). However, most of the countries that cultivate GM crops do not have a lot of published articles on this subject and this review identifies and engages with the legislation in place in those countries, the ongoing discussions on gene edited crops and the related challenges.

### Defining a Genetically Modified Organism

So, what then, is a genetically modified (GM) crop and why are we renewing the discussion on regulating the products of biotechnology? Words matter in law, so we begin with the definition: the definition of a GMO or GM crop is contained in the United Nations (UN) Cartagena Protocol on Biosafety^1^ of a “living modified organism” (LMO). A plant is genetically modified if it meets two requirements: (1) the plant contains a novel combination of genetic material, (2) which was introduced by using modern biotechnology^2^. “Modern biotechnology” is legally defined as an application of either *in vitro* nucleic acid techniques (which includes recombinant DNA and direct injection of nucleic acid into cells or organelles) or the fusion of cells beyond the taxonomic family^3^.

At the time of drafting of the Cartagena Protocol in the early 2000’s, the legal definition of modern biotechnology was a means of clearly separating certain biotechnology techniques from those that are considered more traditional, namely plant breeding, selection and conventional mutagenesis techniques (Mackenzie et al., 2003). For a full report on the variety of plant breeding methods that can be employed without triggering national GMO regulations for commercialization, see van de Wiel et al. (2010). Creation of new phenotypes through “conventional” mutation breeding by use of mutagenic chemicals or radiation has given rise to more than 3,300 registered varieties in over 240 plant species. Although plants developed using mutation breeding meet the first requirement of the definition of a LMO/GMO (it contains a novel combination of genetic material), it is excluded because it was a method regularly used *before* the introduction of recombinant DNA methods [see Bado et al. (2015) for a general overview and the Mutant Variety Database (FAO IAEA, 2020) for updated details on this topic which is beyond the scope of this review article].

The GMO definition contained in the Cartagena Protocol is the international guiding definition for individual states and their governments to utilize in their biosafety legislation. As will be discussed in this review, most countries reflect the above definition or a close variation thereof in their legislation governing GM crops. The resulting biosafety legislation of the individual countries determines the risk assessment and management strategies for the release and commercialization of the GM crop. Herein lies the diversity across the globe and which this review aims to synthesize for readers as a reference point.

### A Renewed Discussion Amongst Regulators

The latter question, concerning the renewal on the discussion on regulating biotech plants, involves how legislators will come to define new breeding technologies (NBTs) such as gene editing – is it “genetic modification”? Gene editing, or genome editing, is the use of site-directed nucleases (SDN) to make exceptionally precise incisions at the target DNA area (Metje-Sprink et al., 2019). There are currently five tools that may be employed for gene editing purposes: (1) Oligonucleotide Directed Mutagenesis (ODM) (Wallace et al., 1981), (2) Zinc-Finger Nucleases (ZFNs), (3) meganucleases, (4) Transcription Activator-Like Effectors Nucleases (TALENs), and (5) Clustered Regularly Interspaced Short Palindromic Repeats (CRISPR) systems (Puchta, 2017; Metje-Sprink et al., 2019). Regulators currently rely on the broader categorization of these technologies as SDN-1, SDN-2, and SDN-3, the classification introduced by Lusser et al. (2011, 2012).

Briefly, for purposes of better understanding where regulators are placing the threshold for definition as a GMO, the SDN categories describe the following induced changes to the plant DNA: the SDN-1 technique guides the nuclease to a specific site of the DNA to induce a single double-stranded break (DSB) or two DSBs to delete a part of the DNA. The single DSB is repaired by the natural plant mechanisms to create a random mutation. The SDN-2 technique utilizes a small donor DNA template to guide the repair of the DNA to give rise to the desired mutation sequence. Finally, the SDN-3 technique uses a much longer donor DNA template that is then inserted into the target site, much the same result as using traditional recombinant DNA technology (Podevin et al., 2013).

Scientists aver that gene editing is not “genetic modification” because the method of introducing changes to the DNA is no different from changes that can occur during conventional breeding or in nature (NASEM, 2016; Pacher and Puchta, 2017). Crop varieties with the same phenotypes can be created either based on induced random mutagenesis (e.g., mutation breeding) or gene editing technology (e.g., CRISPR/Cas9) (Holme et al., 2019). For example, powdery mildew-resistant wheat based on *mlo*-knockouts have been created based on targeted selection of induced mutations (Acevedo-Garcia et al., 2017) and gene editing (Wang et al., 2014). Although the resulting varieties in these examples are phenotypically indistinguishable, they will in most countries be subjected to completely different legislation, as shown later in this review. Based on the potential for developers to use NBTs to create improved crops that may be able to avoid the onerous regulatory assessments associated with GM crop commercialization, the shift to gene editing technologies is tempting (Pacher and Puchta, 2017; Kumlehn et al., 2018; Sedeek et al., 2019).

## An Overview of the Global Legislative Landscape

In the last 2 years, 26 countries grew around 190 million hectares of biotech crops, almost equally split between 21 developing countries and five industrial countries. Industrial countries include the United States, Canada, Australia, Spain, and Portugal, growing ∼46% of the of the total biotech crops. Of the ∼54% grown by developing countries, Brazil, Argentina, and India are amongst the top five countries with the largest area of biotech crop cultivation (ISAAA, 2018, 2020b). The question is then: what is the regulatory standpoint in these countries that enables cultivation of GM crops on such a large scale?

Broadly, when regulating biotech crops, there is a distinction between approvals for cultivating GM crops, for import and export, and consumption of GM food and feed products. The distinction exists because of the different risks associated with cultivating, trading, and consuming, requiring different regulatory approaches. Often, several official bodies are involved in the assessment of the approval request. For example, in the United States, depending on the nature of the final product, it could fall within the purview of either the United States Department of Agriculture (USDA), the Environmental Protection Agency (EPA), or the Food and Drug Administration (FDA), or more than one agency.

Universally, government legislators strive to enact regulations that protect its citizens, society and the environment. Likewise, regulations applicable to plants and crops destined for food, feed and industry are centered around these aims. How this is achieved is country or region dependent. Commonly, the GM regulations are categorized as either process or product oriented (Callebaut, 2015; Medvedieva and Blume, 2018; Eckerstorfer et al., 2019). Process-oriented regulations regard GM technologies as a novel technique compared to conventional methods, thus, triggering specific legislation to be applied. The emphasis is on the process used to produce the novel product. The product-oriented regulations, however, emphasize the novel characteristics of the product in comparison to those produced by conventional breeding (McHughen, 2016). Thus far, Canada remains the only country which has based their entire GM legislation on the product, rather than the process.

The discourse has centered around determining which system is best suited for the regulation of products developed using gene editing techniques (Kuzma, 2016). Eckerstorfer et al. (2019) determined that both systems exhibit their own advantages and disadvantages without one system being superior over the other. However, biotechnology scientists around the world would generally support the product-based review process as the more scientific approach (Scheben and Edwards, 2018). As McHughen (2016) argues, science and scientific assessments form the basis of effective risk management, and it is risk management which regulations rely on to protect society and the environment. Thus, science must shape regulatory policies, although this cannot, and does not, occur in isolation.

### The Restrictive European Regulatory Approach

#### European Union

In the European Union (EU), Regulation (EC) No 1829/2003 on genetically modified food and feed automatically binds all 27 Member States and specifically concerns GM food and feed produced “from” a GMO^4^. The Regulation aims to ensure that the authorization procedures concerning GM food and feed achieve a high level of protection to human, animal and environmental health^5^. This Regulation applies specifically to food and feed products and their imports, in conjunction with Regulation 1830/2003 regarding tracing and labeling of GM products^6^. Cultivation of GM crops, on the other hand, is the choice of Member States via Directive 2001/18/EC on the deliberate release into the environment of genetically modified organisms (sometimes referred to as the “Cultivation Directive”) (Table 1). This latter instrument specifically provides for the cultivation of GM crops and plants following a rigorous assessment of potential adverse effects on human health and the environment^7^.

**TABLE 1:** Europe – regulatory documents for commercial release of GM crops and status of legislation on gene edited plants.

Country	GM commercial cultivation	Legislation on release of GM crops	Links to GM legislation	Gene editing legislation	Link to GE legislation	Academic references
European Union	Only Spain and Portugal	Regulation (EC) No 1829/2003 of the European Parliament and of the Council of 22 September 2003 on genetically modified food and feed	https://tinyurl.com/y9yn2p8x	Decision of the ECJ, but report and proposal requested from EU Commission (due 30 April 2021)	https://ec.europa.eu/food/plant/gmo/modern_biotech/new-genomic-techniques_en	Jorasch, 2020; Menz et al., 2020
		Directive 2001/18/EC of the European Parliament and of the Council of 12 March 2001 on the deliberate release into the environment of genetically modified organisms and repealing Council Directive 90/220/EEC	https://tinyurl.com/y82dhdrk			
Norway	None	Gene Technology Act of 2 April 1993 No. 38 relating to the production and use of genetically modified organisms, etc	https://www.regjeringen.no/en/dokumenter/gene-technology-act/id173031/	None, but proposal submitted		Borge, 2018; Bratlie et al., 2019
Russian Federation	None	Federal Law of 3 July 2016 No. 358-FZ “On amendments to certain legislative acts of the Russian Federation concerning improvement of the state regulation in the sphere of genetic-engineering activities”	http://publication.pravo.gov.ru/Document/View/0001201607040147?index=0	None		
Switzerland	None	Federal Act on Non-Human Gene Technology (Gene Technology Act, GTA) of 21 March 2003 (Status as of 1 January 2018)	https://www.admin.ch/opc/en/classified-compilation/19996136/index.html	None		

Through “the Cultivation Directive” (Hundleby and Harwood, 2019), Member States can choose to “provisionally restrict or prohibit the use and/or sale of that GMO as or in a product on its territory”^8^. Hence, if the EU body approves a certain GM crop for cultivation, Article 23 enables Member States to restrict or prohibit the cultivation of that GM crop in all, or part of their territory. Since the introduction of the safeguard clause in 2015, several EU countries or regions have prohibited cultivation of GM crops, creating a *de facto* ban on cultivation (Lombardo and Grando, 2020). Of the two events approved for cultivation in the EU in the last 25 years, only one event, an insect-resistant maize (MON810) is routinely cultivated in Spain and Portugal (ISAAA, 2018).

The definition of “genetically modified organism” followed by the EU is often held up as the example of a process-triggered regulatory scheme (Marchant and Stevens, 2015; Sprink et al., 2016; Eckerstorfer et al., 2019). Article 2(2) of the Cultivation Directive deems an organism genetically modified if the method of altering genetic material is done in a way that is not natural mating and/or recombination. In 2018, the European Court of Justice (ECJ) delivered its finding that organisms altered by means of site-directed mutagenesis like CRISPR/Cas9 was included in the definition of a GMO (ECJ, 2018, para 54). As Wasmer (2019) points out, the ramification of the judgment is that the size or type of alteration to the genetic material is irrelevant – if there is mutagenesis, *random* or directed, big or small, the organism is legally deemed a GMO. The ECJ thus clarified that this rule is the point of departure but that the accompanying exceptions^9^ in the Cultivation Directive were included on the basis of their long safety record (an element of time and experience) (ECJ, 2018 para 44–46, 48–53).

The EU legislation catches most plant products that have been modified, aside from those created by the exempted techniques, which includes mutation breeding based on techniques that were in use before the Directive entered into force in 2001, but not newer forms of mutagenesis (Eriksson et al., 2020). For a detailed review of the ECJ judgment on the interpretation of exempted techniques, refer to Purnhagen et al. (2018); Wanner et al. (2019), and Wasmer (2019). The interpretative result can only be described as arbitrary, a result often arising when the legislative instrument provides little deviation from the letter of the law. A further discussion of characteristics like flexibility and certainty of the law proceeds in section “Discussion” of this review. In other words, when arbitrary decisions arise, the implication is that those regulations are no longer fit for purpose (Smyth and Lassoued, 2019; Eriksson et al., 2020; Jorasch, 2020). The standpoint of the EU vastly influences countries exporting to Europe, such as the former European colonies (Paarlberg, 2010, 2014).

In light of the decision by the ECJ, the Council of the European Union requested a study and proposal on the status of “new genomic techniques” to be submitted by April 2021^10^ (Table 1). This is a step in the right direction, where concrete evidence and regulatory practices will underpin the evolution of the law. The European Network of GMO Laboratories (ENGL) has already published their report on detecting food and feed products created by NBTs, identifying various possibilities and challenges (ENGL, 2019). At this stage, the EU relies on the GM legislation for products entering the country and thus the onus is on the developer of the gene edited product to provide the functional detection method of their product but none such products have been submitted for market authorization (European Commission, 2019). Since the EU imports most of their GM products, the study seems glaringly focused on gene edited food and feed products and not so much on cultivation, which is identified as an objective among its other objectives (European Commission, 2019).

#### Non-EU Countries

Norway and Switzerland both restrict the cultivation of GM crops in their national legislations (Table 1). A wide difference exists in their respective approaches to restricting GM crops. Switzerland maintains a temporary moratorium on cultivation and processing of GM crops since 2006, extended until 2021, but continues to import for animal feed purposes Federal Office for the Environment (FOEF, 2018; Table 1). Nevertheless, in 2016, when the moratorium was extended for the third time, the Swiss Cabinet included a recommendation for the creation of separate GM crop zones from 2021, depending on farmer interest. By proposing a coexistence of GM crops from conventional agriculture, the cabinet wishes to foster greater acceptance of GMOs and to leave the door open for their future employment (Chandrasekhar, 2016).

Conversely, Norway sees no cultivation and no import of GM food or feed crops to date but GM crops are legally permitted by the Gene Technology Act (Table 1). The Norwegian Food Safety Authority has not yet approved any products or their deliberate release, except a single species of ornamental purple carnations (Mattilsynet, 2020; ISAAA, 2020a). In addition to the health and environmental safety criteria followed by the EU, Norwegian law further demands the assessment of three non-safety categories: societal benefit, sustainable, and ethically sound products^11^. As relatively broad categories for interpretation, the focus of the three categories lies primarily on the growers and producers of GM crops in developing countries and only partly on the Norwegian consumer (Rosendal and Myhr, 2009). The content and interpretative challenges of these three relatively broad categories is discussed in Rosendal (2008) and Rosendal and Myhr (2009).

Despite the Norwegian government’s ever-strict stance on GM products, the Norwegian Biotechnology Advisory Board recently published and delivered a proposal to the government for the relaxation of legislation concerning deliberate release of GMOs (Borge, 2018; Bratlie et al., 2019). One of the principle motivators for the publication was to address the criticism that the EU regulations are no longer fit for purpose. Rather, the Advisory Board identifies nuances in the application of biotechnology, proposing a nuanced regulatory framework made up of tiers, in an effort to bridge the gap between science and law.

Similarly, the Russian Federation prohibited the cultivation of GM plants and breeding of GM animals under the amendments in Federal Law No. 358-FZ in July 2016 together with the recent approval of the new Food Security Doctrine in January 2020^12^ (Table 1). The amendments are much like those in the EU, where cultivation is prohibited but imports of approved GM food and feed can continue (USDA FAS, 2016), despite media headlines to the contrary (The Moscow Times, 2016). Influenced by the public anti-GMO campaign and strongly supported by the Minister of Agriculture (Galata Bickell, 2019), the new prohibitive position puts an end to the anticipated start of cultivation in 2023 and 2024 (USDA FAS, 2016).

### North America as a Global Cultivator

The United States is considered the global leader in the development and commercialization of GM crops, holding close to 30% of the global market share in Agricultural Biotechnology (Report Linker, 2020). Unlike most countries, the United States has no specific overarching federal law targeted at regulation of genetically modified organisms. Instead, newly developed GM products are directed to specialized regulatory bodies under the Coordinated Framework for Regulation of Biotechnology (Table 2). This means that GM products are assessed under the health, safety and environmental laws that also apply to conventional products, so that similar products can be treated similarly by the appointed agencies^13^.

**TABLE 2:** North America – regulatory documents for commercial release of GM crops and status of legislation on gene edited plants.

Country	GM commercial cultivation	Legislation on release of GM crops	Links to GM legislation	Gene editing legislation	Link to GE legislation	Academic references
Canada	Yes	Directive 94-08 (Dir 94-08) Assessment Criteria for Determining Environmental Safety of Plants With Novel Traits	https://tinyurl.com/yu2chsqy	Same as cultivation legislation		McHughen, 2016; Smyth, 2017
United States	Yes	Coordinated Framework for Regulation of Biotechnology, 51 Fed. Reg. 23, 302 (June 26, 1986)	https://www.aphis.usda.gov/brs/fedregister/coordinated_framework.pdf	SECURE Rule	https://www.aphis.usda.gov/aphis/ourfocus/biotechnology/biotech-rule-revision/secure-rule/secure-text/sr-text	Wolt and Wolf, 2018
		Title 7 Code of Federal Regulations (CFR) Part 340 Introduction of organisms and products altered or produced through genetic engineering which are plant pests or which there is reason to believe are plant pests	https://www.law.cornell.edu/cfr/text/7/part-340			

Assessment of novel GM crop plant products can occur under a variety of legislation and agencies, including the FDA, EPA, and USDA. Specifically, the USDA’s Animal and Plant Health Inspection Service (APHIS) is mandated to oversee that introduction of GM plants do not pose a pest risk to plants (Table 2). The plant product either receives regulated or non-regulated status, the latter status allowing cultivation, import and transport without regulatory oversight by APHIS. It is crucial to point out that non-regulated status by APHIS only encompasses the introduction of the GM plant for cultivating and transport. If the GM plant is intended for food use, the FDA holds the mandate to assess the safety of the GM food product. At the time of writing, 128 GM plant varieties received non-regulated status because they do not contain foreign DNA from “plant pests,” including bacteria, fungi, viruses, insects, etc (USDA APHIS, 2020a). This is also true of CRISPR/Cas9-modified food crops, when in 2016, a common button mushroom (*Agaricus bisporus*), modified to resist browning and thus reduce spoilage, was granted non-regulated status (Waltz, 2016). Since then, several gene edited products have already entered the market: Calyno^TM^, a high oleic soybean oil, SU (sulfonylurea) Canola^TM^, a herbicide tolerant canola and a waxy corn (Lassoued et al., 2019; USDA APHIS, 2020b). For an in-depth analysis of the regulations applicable to genome editing in the United States, see Wolt and Wolf (2018) (Table 2).

Canada also features in the top five largest biotech crop cultivators, accounting for approximately 6.6% of the total global biotech crop area in 2018 (ISAAA, 2018). It is noteworthy that Canada follows the product-oriented approach in their legislation, which, some argue, fosters innovation in agricultural biotechnology (Atanassova and Keiper, 2018; Whelan et al., 2020). What distinguishes Canadian legislation from other product-based regulatory schemes is the mere presence of a novel trait, not the way it was introduced. Whether the novel trait was developed by conventional breeding techniques, traditional mutagenesis, or targeted mutagenesis, the novel plant product is subject to the same risk assessment regulations Canadian Food Inspection Agency (CFIA, 2020; Table 2).

Smyth (2017) argues that Canada, particularly, has maintained a strictly science-based assessment of risks when it comes to novel plants, focusing on allergenicity, toxicity and off-target impacts of the product. The regulations are triggered when a specific trait in the plant expresses at least 20–30% lower or higher than the conventional varieties. The plant is then categorized as a plant with novel traits (referred to as PNT) and not a “GMO” (CFIA, 2020). All applications for commercialization must be submitted to the Canadian Food Inspection Agency (CFIA) for unconfined environmental release. Plant products intended for food must additionally undergo an assessment by Health Canada and an assessment of feed by the Animal Feed Division of the CFIA (Government of Canada, 2020).

The unique approach of Canada is best described with an example. Falco^TM^ Canola (Cibus Canola Event 5715) produced by Cibus Canada Inc (Cibus Canada Inc., 2020), is an herbicide tolerant canola, created by employing a NBT, an oligonucleotide-directed mutagenesis (ODM), causing a single nucleotide mutation in two genes. The ODM technique is considered a gene editing technique similar to CRISPR/Cas9. The Government of Canada determined in 2013 that the novel canola variety was no different from unmodified (conventional) canola varieties, determining it as a non-GM crop Canadian Food Inspection Agency (CFIA, 2013; Health Canada, 2013).

### Striving for Legislative Uniformity in Latin America

Both Brazil and Argentina occupy spots in the top five GM cultivating countries. Together with Bolivia, Chile, Colombia, Costa Rica, Honduras, Mexico, Paraguay and Uruguay, Latin America cultivated a staggering 42.7% of the global GM crop area (ISAAA, 2018). There has also been an incredible move to harmonize the regulations concerning GM products in South America. In 2017, the Ministers of Agriculture from Argentina, Brazil, Chile, Paraguay, and Uruguay signed a declaration^14^ on new breeding techniques that specifically recognizes and strives to reduce inconsistent approvals across the region (Norero, 2018; Benítez Candia et al., 2020). In the last 5 years, eight out of 12 Latin American countries have drafted documents for this purpose. The overarching policy is one of assessment on a case-by-case basis, providing opportunities for certain gene edited products to be excluded from strict regulation (Table 2; Whelan and Lema, 2015; Gatica-Arias, 2020).

Opposition to GM crops remains in Ecuador, Venezuela, and Peru, who do not permit commercial cultivation of GM crops. In 2008, Ecuador enacted its Constitution, enshrining that Ecuador is “free of transgenic crops and seeds.” The President may, if he/she deems it in the interest of the nation, condone the introduction of GM seeds into the country^15^. Relying on this exception, the Ecuadorian government enacted legislation allowing the entry and cultivation of GM seeds for research purposes only (Table 3; Norero, 2017; Gatica-Arias, 2020). Despite the transgenic-free declaration, Ecuador is a recent addition to the group of countries focused on harmonizing policy to accommodate new breeding techniques by implementing Executive Decree No. 752 in May 2019 (Table 3). Article 230(a) excludes those organisms that do not contain foreign or recombinant DNA from a risk assessment that would normally apply to GM organisms (Table 3; Gatica-Arias, 2020).

**TABLE 3:** Latin America – regulatory documents for commercial release of GM crops and status of legislation on gene edited plants.

Country	GM commercial cultivation	Legislation on release of GM crops	Links to GM legislation	Gene editing legislation	Link to GE legislation	Academic references
Argentina	Yes	Law on Seeds and Phytogenetic Creations, Law 20247 (1973)	http://servicios.infoleg.gob.ar/infolegInternet/anexos/30000-34999/34822/texact.htm	Resolution No 173/2015 of the Secretariat of Agriculture, Livestock and Fisheries and of the Ministry of Agriculture, Livestock, and Fisheries	https://www.argentina.gob.ar/normativa/nacional/resoluci%C3%B3n-173-2015-246978/texto	Lema, 2019; Whelan et al., 2020
		Law on the Promotion of the Development and Production of Modern Biotechnology, Law 26270 (2007)	http://extwprlegs1.fao.org/docs/pdf/arg72993.pdf			
Bolivia	Yes	Law No. 144 – Law of Productive, Communal, and Agricultural Revolution	http://www.fao.org/faolex/results/details/en/c/LEX-FAOC120110	None		
Brazil	Yes	Law No. 11.105 of 24 March 2005	http://www.planalto.gov.br/ccivil_03/_ato2004-2006/2005/lei/l11105.htm	Normative Resolution No 16 – Sets forth the technical requirements for submitting an inquiry to the CTNBio concerning Precision Breeding Innovation Techniques (2018) *Does not change existing regulations	https://tinyurl.com/4se3g2jm	Gatica-Arias, 2020
		Law No. 11.460 Regulating the planting of Genetically Modified Organisms within conservation areas (2007)	https://www.informea.org/en/legislation/law-no-11460-regulating-planting-genetically-modified-organisms-within-conservation			
Chile	Yes, for export only	Exempt Resolution No. 1523 of 2001 establishing rules for the intermation and introduction to the environment of living modified plant organisms of propagation	https://www.bcn.cl/leychile/navegar?idNorma=187630	Publication on Servicio Agrícola y Ganadero (SAG) website on the applicability of Resolution 1523 of 2001 to propagation material developed by new plant breeding techniques *does not change existing regulations	http://www.sag.cl/ambitos-de-accion/aplicabilidad-de-resolucion-ndeg-15232001-en-material-de-propagacion-desarrollado-por-nuevas-tecnicas-de-fitomejoramiento	Salazar et al., 2019; Sánchez, 2020
Colombia	Yes	Regulatory Decree 4525 of 6 December 2005	http://www.suin-juriscol.gov.co/viewDocument.asp?ruta=Decretos/1547044	Resolution 29299 of 2018 “By which the procedure for the processing before the ICA of applications for an improved cultivar with innovation techniques in plant breeding through modern Biotechnology is established, in order to determine if the cultivar corresponds to a Living Modified Organism or a conventional one”	https://www.ica.gov.co/normatividad/normas-ica/resoluciones-oficinas-nacionales/2018/2018r29299	Gatica-Arias, 2020
Costa Rica	Yes	Law No. 7664 – Phytosanitary Protection Law	http://www.fao.org/faolex/results/details/en/c/LEX-FAOC012354	None		Gatica-Arias, 2020
		Decree No. 26.921/MAG – Regulation to the Phytosanitary Protection Law	http://www.fao.org/faolex/results/details/en/c/LEX-FAOC015486			
Ecuador	No, research only	The Organic Law on Agrobiodiversity, Seeds, and Promotion of Sustainable Agriculture (2017)	https://www.gob.ec/regulaciones/ley-organica-agrobiodiversidad-semillas-fomento-agricultura-sustentable	Art. 230(a) Executive Decree No. 752 of 21 May 2019 Regulations to the Organic Code on the Environment	https://s3.amazonaws.com/lexis.news.storage/Mayo%202019/d_752-comprimido_reduce(2)_20190421231939.pdf	Norero, 2017; Gatica-Arias, 2020
Honduras	Yes	Agreement No. 1570/98 Biosecurity Regulation with Emphasis on Transgenic Plants	http://www.fao.org/faolex/results/details/en/c/LEX-FAOC043010	Agreement No. 8-SENASA-2019 Authorization procedures for applications related to the use of new genetic improvement techniques (precision biotechnology)	http://www.fao.org/faolex/results/details/en/c/LEX-FAOC190734	Gatica-Arias, 2020
Mexico	Yes *no new GMO permits approved since May 2018	Law on Biosecurity of Genetically Modified Organisms (text date 18 March 2005)	http://www.fao.org/faolex/results/details/en/c/LEX-FAOC064015	None		
Myanmar	Yes	No biosafety law (under revision). Cultivation occurs in terms of the National Seed Policy, 2016	http://www.doa.gov.mm/doa/index.php?route=product/product/freedownload&freedownload_id=176	None		
Paraguay	Yes	Decree No. 9699/12 by which the National Commission for Agricultural and Forestry Biosafety (CONBIO) is created	http://www.fao.org/faolex/results/details/en/c/LEX-FAOC130178	Resolution No 565 of 13 May 2019 approves the Form of Prior Consultation for products obtained through new techniques of genetic improvement) *link to original resolution not obtained	https://cdn-www.lanacionpy.arcpublishing.com/negocios_edicion_impresa/2019/05/25/facilitaran-uso-de-las-tecnologias-geneticas/	Benítez Candia et al., 2020; Gatica-Arias, 2020
Peru	None – 10 year moratorium expires in 2021	Regulation of Law No. 29811, Law that establishes the Moratorium on Entry and Production of Living Modified Organisms to the National Territory for a Period of 10 years	https://www.minam.gob.pe/wp-content/uploads/2013/08/113252603-reglamento-ley-moratoria-ovm.pdf	None		Branford, 2013; Dondanville and Dougherty, 2020
Uruguay	Yes	Decree No. 353/008 – Biosafety of Genetically Modified Vegetables	https://www.aduanas.gub.uy/innovaportal/v/7531/1/innova.front/decreto-n%C2%BA-353_008.html	None but joint international statement to the WTO in October 2018	https://tinyurl.com/y6bbqsx2	Gatica-Arias, 2020
Venezuela	None	Seed law of Venezuela, 26 June 2018	http://www.biodiversidadla.org/Documentos/Ley_de_Semillas_de_Venezuela2#:~:text=Esta%20ley%20promueve%20el%20desarrollo,%2C%20soberano%2C%20democr%C3%A1tico%2C%20participativo%2C	None		Gómez et al., 2010; Herrera et al., 2017

* indicates special/additional notes to the regulation.

In 2011, Peru enacted a 10-year legislative moratorium on GM crops, banning the entry and cultivation of GM seeds (Table 3; Branford, 2013). As the expiration date approaches in 2021, the Peruvian Congress approved the extension of the moratorium another 15 years. However, the extension is not yet official without the signature of the President, a position currently in political flux (Montaguth, 2020). Although Dondanville and Dougherty (2020) argue that the moratorium was merely a means to create space for the government to enact regulations that would pave the way for adoption of agricultural biotechnology, it is clear that the government of Peru has no strategies in place for regulating gene edited products either (Gatica-Arias, 2020).

Similarly, Venezuela enacted the Seed Law in 2015, prohibiting all GM plants and seeds, including those for research purposes (Table 3; APBREBES, 2016; Global Agriculture, 2016). According to the strategy document for conservation actions, the introduction of GMOs is identified as one of four direct causes of loss of biodiversity in Venezuela (Gómez et al., 2010). One of the overarching concerns driving the prohibition on environmental release is the “agroecology” of Venezuela (Herrera et al., 2017). Yet, Venezuela remains significantly reliant on food and feed imports of GM soybean and maize crops from neighboring Brazil, Argentina, and the United States (USDA FAS, 2018).

The framework governing GM and gene edited plants in Chile has developed in an unusual manner. On the one hand, regulators were quick to implement a case-by-case approach for plants developed using new breeding techniques, the second country after Argentina to do so (Table 3). The consultation process with the Agricultural and Livestock Service (SAG) body determines whether the variety or product is considered a GMO or not, based largely on whether there is presence of foreign DNA (Sánchez, 2020). Thus far, eight products have been deemed non-GMO and can be released as conventionally bred plants (Eriksson et al., 2019; Sánchez, 2020). However, if the plant is designated as GMO, and for traditional GM plants (i.e., not developed by new breeding techniques and falls within the definition of a “GMO”), no comprehensive biotechnology framework exists (Table 3; USDA FAS, 2020a).

Despite the absence of a complete regulatory framework for GM plants, SAG plays an integral role in the stringent control of reproduction of GM seeds for the export market. Chile ranks ninth in the world for seed exports, a large part of which are GM seeds, making Chile the “southern seed nursery for the GM industry” (Salazar et al., 2019; International Seed Federation – ISF, 2020). To this end, SAG relies on Resolution 1523 of 2001 to monitor and control GM seed import, production, field trials and export of GM seeds (Table 3). Yet, there is no legislation on the domestic use of these GM seeds for food and feed, meaning that GM seeds are not allowed to be cultivated in the country as domestic product (Salazar et al., 2019). Remarkably, no restrictions exist to import GM food and feed grown in other countries, with most of their soybean and maize imports coming from Brazil (Sánchez and León, 2016).

### Little Known Regulatory Landscape in Africa

Despite considerable challenges to food security from population growth and climate, a handful of the 47 countries in Africa currently cultivate GM crops: Ethiopia, Kenya, Malawi, Nigeria, South Africa, Sudan and recently, eSwatini (formerly Swaziland) (Table 4). South Africa, as the first African country to enact a regulatory framework to allow GM crop cultivation, import and export, is also the largest GM crop producer in Africa with the ninth largest biotech crop area globally (Table 4; ISAAA, 2018). Although no commercial cultivation currently takes place in Burkina Faso, the regulations to do so remain open under the Biosafety Law of 2012 (Table 4).

**TABLE 4:** Africa – Regulatory documents for commercial release of GM crops and status of legislation on gene edited plants.

Country	GM commercial cultivation	Legislation on release of GM crops	Links to GM legislation	Gene editing legislation	Link to GE legislation	Academic references
Burkina Faso	Permitted but no current commercial cultivation	Law No. 064-2012 Instituting a Biotechnology Security System/Biosafety Law of 2012	https://tinyurl.com/ujg9q36y	None, but a regional biosafety law for the Economic Community of West African States (ECOWAS) community is under revision	https://www.ecowas.int/ecowas-moves-to-ensure-biosafety-in-the-region/	Dowd-Uribe and Schnurr, 2016; Gakpo, 2020
Egypt	Suspended by Ministerial Decree 378/2012	No Biosafety Law. National Biosafety Commission established under Ministerial Decree 1495/2014 but remains dormant since 2014	https://apps.fas.usda.gov/newgainapi/api/Report/DownloadReportByFileName?fileName=Agricultural%20Biotechnology%20Annual_Cairo_Egypt_10-20-2020	None		Gakpo, 2019
eSwatini	Yes	Biosafety Act, 2012 (No. 7 of 2012)	http://extwprlegs1.fao.org/docs/pdf/swa195286.pdf	None		Muzhinji and Ntuli, 2021
Ethiopia	Yes	Biosafety Proclamation No. 655/2009	http://extwprlegs1.fao.org/docs/pdf/eth95180.pdf	None		Gebretsadik and Kiflu, 2018; Tsegay, 2019
		Amended by Biosafety (Amendment) Proclamation No. 896/2015	https://www.efccc.gov.et/images/Proclamation/Proclamation-No-896-2015-Biosafety-amendment.pdf			
Ghana	None	Biosafety Act, 2011 (Act 831)	http://extwprlegs1.fao.org/docs/pdf/gha136733.pdf	None, but a regional biosafety law for the Economic Community of West African States (ECOWAS) community is under revision	https://www.ecowas.int/ecowas-moves-to-ensure-biosafety-in-the-region/	Adenle et al., 2013; Komen et al., 2020
Kenya	Yes, since 2020	Biosafety Act, 2009	http://kenyalaw.org:8181/exist/kenyalex/actview.xql?actid=No.%202%20of%202009	Draft guidelines on genome editing		Komen et al., 2020
		Implemented by: The Biosafety (Environmental Release) Regulations, 2011	http://www.biosafetykenya.go.ke/index.php?option=com_content&view=article&id=17&Itemid=122			
Malawi	Yes	Biosafety Act, 2002 (Chapter 60:03) implemented by Biosafety (Management of Genetically Modified Organisms) Regulations, 2007	https://malawilii.org/mw/consolidated_legislation/6003	None		Muzhinji and Ntuli, 2021
Nigeria	Yes, since 2018	National Biosafety Management Agency Act, 2015	https://lawnigeria.com/2019/11/national-biosafety-management-agency-act-2015/	National Biosafety Management Agency (Amendment) Act, 2019 enlarges scope of NBMA Act to include new breeding techniques as GMO.	https://lawnigeria.com/2019/12/national-biosafety-management-agency-amendment-act-2019/	Komen et al., 2020
		National Biosafety (Implementation, etc.). Regulations 2017	https://www.aatf-africa.org/wp-content/uploads/2020/01/Nigeria-Biosafety-Regulations-2017.pdf			
South Africa	Yes	Genetically Modified Organisms Act, 1997 (No. 15 of 1997)	http://www.saflii.org/cgi-bin/disp.pl?file=za/legis/num_act/gmoa1997286/gmoa1997286.html&query=gmoa1997286	None, but report by Academy of Science of South Africa (ASSAF) published: The Regulatory Implications of New Breeding Techniques (NBTs) (2017)	https://research.assaf.org.za/bitstream/handle/20.500.11911/29/2017_%20assaf_new_breeding_techniques.pdf?sequence=5&isAllowed=y	Pillay and Thaldar, 2018; Agaba, 2019
Sudan	Yes	National Biosafety Law No. 15 of 2010	http://www.fao.org/faolex/results/details/en/c/LEX-FAOC150548	None		Agaba, 2019
Uganda	None	National Biosafety Act, 2017 pending Presidential assent	http://parliamentwatch.ug/wp-content/uploads/2015/03/The-national-biotechnology-and-biosafety-bill-2012-1.pdf	None		Zawedde et al., 2018; Komen et al., 2020

In his book, Schnurr (2019) covers the historical, political and scientific developments related to traditional GM crops and their regulation in Africa. The author provides an interesting categorization of the regulatory responses in Africa: the early adopters (South Africa, Egypt, and Burkina Faso), the emerging adopters (Uganda, Ghana, Nigeria, Cameroon, Ethiopia, Malawi, Mozambique, and eSwatini), the resisters (Zambia, Zimbabwe, and Tanzania) and the renegades (Kenya and Sudan). When it comes to new breeding techniques and the corresponding regulations, African countries are collaborating and discussing harmonization tactics (Table 4) African Biosafety Network of Expertise (African Biosafety Network of Expertise – ABNE, 2019; Isaac, 2019).

South Africa remains the only African country to approve a GM staple food crop for direct consumption – white maize. Egypt and Burkina Faso initially approved the cultivation of Bt cotton and Bt maize, respectively. However, in 2012, Egypt suspended the planting of GM crops (Gakpo, 2019) and Burkina Faso in 2016 (Dowd-Uribe and Schnurr, 2016; Table 4). Various researchers have traversed the causes for the limited adoption of GM crops (Adenle et al., 2013; Mabaya et al., 2015; Kargbo et al., 2020; Luna, 2020) and evidently, there are strong arguments in this regard. Paarlberg (2009, 2010, 2014) has consistently laid the blame at the feet of prosperous global North countries and their outspoken anti-GMO groups. Yet others contend that there is greater complexity for the slow GM crop adoption in Africa, encompassing social, political, legislative, and business conditions (Scoones and Glover, 2009; Komen et al., 2020; Rock and Schurman, 2020).

Nevertheless, environmental release approvals have recently been granted for GM cotton in Ethiopia, Kenya, Malawi, and Nigeria. Farmers in Ethiopia started planting in 2019 and seed distribution is expected in 2020 in the latter countries (Komen et al., 2020). Likewise, Ghana and Uganda are taking steps to move their field trials to approval for commercial cultivation and Burkina Faso wishes to do the same with Bt cowpea (Gakpo, 2020; Komen et al., 2020). Movements and discussions like these are positive indicators of wider acceptance of traditional GM crops and even more so, plants developed using new breeding techniques, but a fine line must be tread to avoid overregulation that may stifle the progression of innovation (Table 4; Qaim, 2020; Smyth, 2020).

### Asia and the Pacific

#### India and China as Top GM Cotton Producers

Commercial cultivation of GM crops in Asia and the Pacific is permitted in the following countries, in order of area: India, China, Pakistan, Australia, Philippines, Myanmar, Vietnam, Bangladesh, and Indonesia (Table 5; ISAAA, 2018). India is both the world’s largest cotton producer and largest Bt cotton producer with an adoption rate of 95% for Bt cotton (ISAAA, 2018; Shahbandeh, 2020). In 2001, thousands of small-scale Indian farmers were discovered to be illegally planting Bt cotton, before government approval followed in 2002, a typical bottom-up^16^ development of the law (Ramaswami et al., 2012). Although cultivation approval exists for non-food GM cotton, a *de facto* moratorium endures for the GM food crop, Bt brinjal (Kumar et al., 2011). In 2010, the Minister of Environment and Forestry rejected the approval recommendation of the Genetic Engineering Approval Committee (GEAC) (Table 5), bringing about a “temporary” moratorium that continues (Cao, 2018). Nevertheless, there are reports of illegal plantings of not only Bt brinjal (Todhunter, 2019; Blakeney, 2020), but also of stacked IR and HR cotton and virus-resistant papaya (Rao, 2013). The possibility remains for another bottom-up change to the moratorium should farmers in India continue illegal planting of Bt brinjal.

**TABLE 5:** Asia and the Pacific – regulatory documents for commercial release of GM crops and status of legislation on gene edited plants.

Country	GM commercial cultivation	Legislation on release of GM crops	Links to GM legislation	Gene editing legislation	Link to GE legislation	Academic references
Australia	Yes	Gene Technology Act 2000, implemented by Gene Technology Regulations 2001	https://www.legislation.gov.au/Details/C2016C00792	Gene Technology Regulations 2001	https://www.legislation.gov.au/Details/F2020C00957	Kelly, 2019; Thygesen, 2019
Bangladesh	Yes	Bangladesh Biosafety Rules, 2012	https://bangladeshbiosafety.org/2017/05/16/bangladesh-biosafety-rules-2012/	None		Schmidt et al., 2020
		2017 User’s Guide to Biosafety Regulatory Process for Genetically Engineered Plants in Bangladesh	http://bangladeshbiosafety.org/wp-content/uploads/2017/11/Users_Guide_to_Biosafety_Regulatory_Process_Bangladesh_2017.pdf			
China	Yes	Regulation on Administration of Agricultural Genetically Modified Organisms Safety	https://www.wipo.int/edocs/lexdocs/laws/en/cn/cn133en.pdf	None		Cao, 2018; Chen and Dai, 2020; Zhang et al., 2020
India	Yes	Environment (Protection) Act, 1986	http://extwprlegs1.fao.org/docs/pdf/ind21695.pdf	None, but Draft Guidelines for Safety Assessment of Genome Edited Plants submitted to the Genetic Engineering Appraisal Committee	http://geacindia.gov.in/Uploads/MoMPublished/MoMPublishedOn20200910170648.pdf	Ramaswami et al., 2012; Cao, 2018; Fernandes, 2020
		Implemented by: Rules for the Manufacture, Use, Import, Export and Storage of Hazardous Micro-Organisms Genetically Engineered Organisms or Cells of 1989	http://extwprlegs1.fao.org/docs/pdf/ind10951.pdf			
Indonesia	Yes, but not full commercial release	Regulation 36/2016 (link to original Indonesian text)	https://www.gpmt.or.id/view/TWluaXN0ZXIgb2YgQWdyaWN1bHR1cmUgUmVndWxhdGlvbiBuby4gMzYvMjAxNg%3D%3D/16c7529d8abdd0c3a0288254715755ac.pdf	None, but discussions continue	https://biotek.lipi.go.id/2020/01/15/teknologi-genome-editing-untuk-kualitas-tanaman-pangan-lebih-baik/	
		Government Regulation of the Republic of Indonesia Number 21 of 2005 concerning the Biosafety of Genetically Engineered Products (original Indonesian text)	https://peraturan.bpk.go.id/Home/Details/49379/pp-no-21-tahun-2005			
Japan	Yes, ornamentals only	Law Concerning the Conservation and Sustainable Use of Biological Diversity through Regulations on the Use of Living Modified Organisms (Law No. 97 of 2003)	http://www.japaneselawtranslation.go.jp/law/detail/?ft=1&re=2&dn=1&x=0&y=0&co=01&ia=03&ja=04&ky=biological+diversity&page=3	About the Handling of Organisms Produced by the Use of Genome Editing Technology that Do Not Match the Definition of “Genetically Modified Organisms” in the Cartagena Act (link to original Japanese text)	http://www.biodic.go.jp/bch/download/genome/genome_tsuuchi20190208.pdf	Tsuda et al., 2019; Matsushita et al., 2020
New Zealand	None	Hazardous Substances and New Organisms Act 1996	https://www.legislation.govt.nz/act/public/1996/0030/latest/DLM381222.html	Amendment to Regulation Clause 3(1) (b) of the Hazardous Substances and New Organisms (Organisms Not Genetically Modified) Regulations 1998	https://www.legislation.govt.nz/regulation/public/1998/0219/latest/DLM255883.html?search=ta_act%40regulation_H_ac%40rc%40ainf%40anif%40rinf%40rnif_an%40bn%40rn_25_a&p=3	Ishii and Araki, 2017; Fritsche et al., 2018
Pakistan	Yes	Pakistan Biosafety Rules, 2005	https://media.nti.org/pdfs/pakistan_bw_biosafety_rules_1.pdf	None		Babar et al., 2020
Philippines	Yes	DOST-DA-DENR-DOH-DILG Joint Department Circular No. 1, Series of 2016	http://biotech.da.gov.ph/upload/Signed_DOST-DA-DENR-DOH-DILG_JDCs2016.pdf	Joint international statement to the WTO in October 2018; Resolution on New Breeding Techniques is expected in 2021	https://docs.wto.org/dol2fe/Pages/FE_Search/FE_S_S009-DP.aspx?language=E&CatalogueIdList=264756,264006,249321,249267,249180,249148&CurrentCatalogueIdIndex=2&FullTextHash=371857150&HasEnglishRecord=True&HasFrenchRecord=True&HasSpanishRecord=True	Pasquito, 2019

As regards gene edited crops and potential changes to the legislation in India, Ahuja (2018) suggests there is room for regulators to use the existing legislation on a case-by-case basis, on the basis that they are not confined by the definition of “modern biotechnology” as contained in the Cartagena Protocol. In January 2020, the Indian government, through its Department of Biotechnology, published proposed gene editing guidelines for public comments (Table 5). The draft guidelines propose a tiered approach, with an increasing number of assessments for increasing number of changes to the DNA (Fernandes, 2020).

China is the second largest cotton producer in the world (Shahbandeh, 2020) and like India, reflects an adoption rate of Bt cotton around 95% (ISAAA, 2017). Since the very beginning of GM crops, China has promoted biotech research with plenty of investment in a two-pronged effort to ensure food security and world-leading agricultural biotechnology (Cao, 2018). China commenced commercialization in 1990 with a virus-resistant tobacco (Raman, 2017). Since its introduction in 1997, Bt cotton seeds have been well received, the majority of which is now being domestically produced. However, Cao (2018) argues that Bt cotton received swift approval (just 2 years) for several reasons, the primary one being that there were no global controversies surrounding GMOs at the time, unlike Bt rice.

At this stage, of the seven crops approved for cultivation, only Bt cotton and virus-resistant papaya are grown on a large scale in China. To start cultivating new GM crops, the applicant must follow a three-phase trials process encompassing field, environmental release, and preproduction trials (Jin et al., 2019). Thereafter, the applicant may obtain an Agricultural GMOs Safety Certificate (a Biosafety Certificate), issued by the Ministry of Agriculture and Rural Affairs (MOARA)^17^. Yet, even with a Biosafety Certificate, cultivation can be blocked, as is the case of two locally developed Bt rice varieties, GM Shanyou 63 and Huahui-1/TT51-1 (ISAAA, 2020a). Although both varieties received short-term Biosafety Certificates in 2009, which were renewed once to expire in 2019, the Bt rice was never officially cultivated. In a recent about-turn at the end of 2019, a list of 192 GM crops set for biosafety clearance was published for public opinion, including GM soybean and maize (Cremer, 2020; Xiaodong, 2020).

Reflecting the development that occurred for transgenic crops, China has injected huge funding for R&D in CRISPR/Cas technology, encompassing use of other Cas proteins (Cohen, 2019). During the period 2014 to 2017, China accounted for 42% of the CRISPR/Cas-related publications in agriculture (more than double that of the United States), and 69% of patent applications for CRISPR/Cas in agriculture (the United States occupies second place with 19%) (Cohen and Desai, 2019; Martin-Laffon et al., 2019). Notwithstanding, China does not yet have a regulatory framework in place to assess gene edited crops for commercial release with some speculating that China may follow the United States model of assessment (Cohen, 2019), while others suggest the Japanese approach may be more fitting (Zhang et al., 2020; Table 5).

Biofortified Golden Rice (event name: GR2E) is one of the most prominent GM crop examples that still lacks release approval. Golden Rice has a gain-of-function trait to produce vitamin A precursor molecules to address critical vitamin A deficiencies in young children and pregnant women in Africa and South-East Asia (World Health Organization, 2020b). Since the early stages in 2000 (Ye et al., 2000), it took 17 years for a handful of countries to grant approval. Presently, Australia, Canada, New Zealand, the United States and the Philippines allow direct human consumption of Golden Rice but no cultivation (ISAAA, 2020a). The irony is that the Philippines is the only country in the target group of countries to give such approval (World Health Organization, 2020a).

#### Regulatory Updates for Gene Editing in Asia-Pacific

Japan implements an unusual approach to GM crop regulations. In 2018, Japan featured second, after the United States, in the number of approval of GM events for food, feed and cultivation (ISAAA, 2018) and that even though 141 GM events for cultivation were approved by 2020, no GM crop planting actually occurs (except for the ornamental blue rose flower) (Table 5; ISAAA, 2020a; USDA FAS, 2020b). The legislation in Japan requires that cultivation approval be obtained for imported products only ever destined for food, feed or processing purposes. In this way, the authorities have had the opportunity to evaluate the environmental risks associated with that GM crop in the event of spilled GM grain or unintended mixing with conventional, non-GM seeds (Table 5; Matsushita et al., 2020). Like Europe, Japan is one of the world’s biggest importers of GM crops, importing close to 100% of their corn and 94% of their soybean supply (USDA FAS, 2020c).

Both Japan and Australia have taken steps in the last several years to clarify their regulatory regimes concerning gene edited crops and products, with similar regulatory outcomes (Table 5). In Japan, the clarification was provided by means of an interpretation document. According to the interpretation by the Japanese Ministry of Environment, products that do not contain inserted DNA or RNA is not considered a “living modified organism” within the meaning of the Cartagena Law (Table 5). This means that organisms created by means of unguided repair of site-directed nuclease activity, known as SDN-1 organisms, are no longer considered LMOs (Tsuda et al., 2019). In Australian law, clarity came in the form of amendments in 2019 to the Gene Technology Regulations 2001, where a new exclusion was introduced (Table 5). SDN-1 organisms are thus not considered GMOs within the meaning of the Gene Technology Act 2000 Office of the Gene Technology Regulator (OGTR, 2020). Practically, this means that the crop no longer falls within the regulatory purview of the Gene Technology Act. Rather, it is directed to regulations under the Department of Agriculture, Water and the Environment and should it produce food products, such products are regulated under the Australia New Zealand Food Standards Code.

Unlike neighboring Australia, New Zealand does not cultivate GM crops and takes a hard line against organisms developed using gene editing techniques. The regulations contained in the 1996 Hazardous Substances and New Organisms Act (HSNO) and administered by the Environmental Protection Authority (EPA) are one of the more comprehensive in the world, with strict minimum standards for approval assessment (Table 5; Fritsche et al., 2018). In the assessment, the EPA must consider whether the benefits of the GMO outweigh the risks and part of that is the impact that the novel plant may have on the Mâori culture and traditions, especially with regards to their valued fauna and flora, ancestral lands, water, sacred places and treasured things (Hudson et al., 2019). More specifically relating to regulation of organisms as a result of new breeding techniques, New Zealand was one of the first countries to amend their legislation to distinguish plants bred by conventional mutagenesis (Table 5). This implies that novel plants created by new breeding techniques, even those without foreign DNA, still fall under the regulations as a GMO (Ishii and Araki, 2017).

## Discussion

### Diversity Informs Harmonization

In the preceding two decades, the reports of cultivation area for biotech crops showed a staggering adoption of GM crops across a diversity of crops. Although this report does not focus on GM crop production numbers, the demand for innovative agricultural tools to combat a range of challenges by farmers and producers remains high. In addition to the GM crops on the market, employment of precise NBTs to breed for desirable crop traits offers the possibility for further customized solutions to the farmer’s demands and which can be developed in a shorter time (Arora and Narula, 2017; Yin et al., 2017). Regulations supporting the flow of gene edited crops onto the market can further cut the time that elapses between the lab and the farmer.

It is clear from the data gathered above that there is a diverse range of legislation and frameworks on how best to regulate GM crop cultivation. Even within continents or larger geographic regions, the local approaches can vary widely, illustrated by the diversity across Asia (See section “Asia and the Pacific” and Table 5). Diversity is also found when there are similar outcomes but different approaches to regulation, as seen in the United States and Canada. Certain frameworks on cultivation do not include trade regulation of GMO products, which is then regulated in a separate document and/or by a separate governmental body. Interestingly, several countries have distinct rules regarding cultivation of GM crops which only allow the production of GM seeds for export and prohibit domestic use. Remarkably, some of these countries then allow for the import of GM crop products as food and feed (see for example Ecuador, Table 3).

In general, the countries which currently dominate the cultivation and export of GM crops have had a framework that is speedy, easy to understand and comply with, and enforceable (Levin, 1994). Although the argument is often that product-based legislation supports the commercialization of GM crops, Ishii and Araki (2017) found this was not the case. Despite their dissimilar process- or product-based approaches, Argentina, Brazil, Chile, Costa Rica, Honduras, Mexico, and Uruguay were some of the first countries in Latin America to provide GM cultivation approvals (Ishii and Araki, 2017; Rosado and Craig, 2017). Today, four of these seven countries are considered biotech “mega-countries” (ISAAA, 2020b). Thus, perhaps there is something more than this trigger of the GMO framework underlying the commercial success of these cultivating countries (Rosado and Craig, 2017). The trend indicates that countries leading in GM cultivation are the same countries that are quickly adapting their biosafety law to accommodate gene-edited products thereby supporting the domestic agricultural sector.

At this stage, it cannot be said that there is harmonization in recognizing that organisms modified by traditional recombinant DNA techniques fall firmly in the category of GMO (see the approach of Canada versus EU and New Zealand). It is the process laid out in the biosafety law that determines whether a GMO crop will reach commercialization or not. Herein lies the globally diverging approaches when regulating GM crops and their related products. Harmonization is the act of making different regulations or standards suitable for others. For most countries that have already implemented an authorization process for gene edited products, harmonization seems to be emerging. Almost all view products created by SDN-1 as not being a GMO and the resultant product will follow the regulatory path of the conventionally bred plant varieties (Schmidt et al., 2020). However, divergence emerges again with regard to SDN-2 techniques: Australia and Japan have opted for a conservative threshold by finding that organisms edited using the SDN-2 technique will be regulated as a GMO (Thygesen, 2019; Tsuda et al., 2019). Such clear differences in the threshold for what constitutes a GMO could frustrate further harmonization efforts.

### Evolving Societal Values Reflected in the Law

As technology rapidly develops in all sectors, including science, we find that law and its interpretation must reflect the values, the *mores*, of the developing societal sector. As Dror (1957) explains, the law is fundamentally an expression of the values of society. The law commands societal obedience by reflecting and expressing the generally accepted social values (Dror, 1957). An example of this are the evolving laws on climate change, which reflect society’s concerns of human impact on the natural world. In today’s society, scientific expertise and analyses plays a greater role than ever before informing societal values and thus causing changes to the law (Lougheed, 2009). Of course, this is an oversimplification to say that scientific experts alone inform the shaping of the law on scientific technology like GM and gene edited crops – there are various competing interests, including bureaucratic, political and societal interests (Lougheed, 2009). The years of scientific, political and regulatory experiences of GM technology and cultivation affords society evidence upon which their values may evolve. This wealth of evidence and experiences are being used to inform the development of laws on gene edited crops. By identifying shared opinions, experiences and technical expertise, harmonization of regulations can be achieved.

During the development of the first biosafety laws, the scientific evidence was sparse, as was the effects that these new crops would have on the environment, diversity and human and animal health (Krattiger and Rosemarin, 1994). After 25 years of field trials, cultivating and trading GM crops, the accumulating bodies of evidence can now further guide and develop the law (Rosado and Craig, 2017). This can be seen with the entry of gene edited crops: the more mature, competent regulatory processes are more flexible in dealing with gene edited products. Additionally, in countries with less experienced regulatory processes (or those without any regulatory oversight), policymakers are being informed and educated by experts in the form of technical advisory bodies and global and regional consultations. The result of which is a call for harmonization of policy in regions across Latin America, North America, and Africa in the shape of statements, declarations and regulations Economic Community of West African States (ECOWAS, 2019; Benítez Candia et al., 2020; Gatica-Arias, 2020).

Argentina, Canada, Australia, and several other countries which have already legislated and implemented their approach to new breeding techniques are not rewriting their GMO law (Atanassova and Keiper, 2018). They are updating and implementing their existing science- and risk-based approaches to assess the products of gene editing technology. Flexibility in their case-by-case basis is argued as one that allows discretion in reaching an outcome CSPM, 2018). In contrast, a law that provides certainty implies a decision without discretion, where there can be no deviation from the letter of the law (Roosevelt, 2019). Flexibility in the application of the law could lend itself to harmonization strategies and still rely on the influx of new scientific evidence on new breeding techniques. It would negate the need to legislate on every single procedure that encompasses new breeding techniques, the situation of “rule and its exceptions,” typical of the traditional distinction in the EU system (where application of recombinant DNA techniques are deemed GMO as a rule, with an accompanying list of technique exceptions).

Even though flexibility is found in the case-by-case approach adopted in several countries when assessing whether a gene edited plant is SDN-1, 2, or 3, the innate character of the framework remains one of “rule-and-exception.” The rule is that gene edited organisms are plants that have undergone a genetic modification requiring an initial assessment on the basis of their creation using NBTs; only those that have been modified without a template or using a small template are categorized as the exceptions. This is not necessarily a negative approach – in fact, it underpins one of the major factors driving the initial evolution of biosafety law: it honors the societal values of risk assessment and risk management for the ultimate goal of preserving human, animal and environmental health.

## Conclusion

The adoption and cultivation of GM crops makes it the fastest growing agricultural technology in the world. Employing complementary new breeding techniques holds promise for providing solutions to food security and changing climate conditions, possibly introducing a wider range and more desirable food products on the market. Regulations on GM crop cultivation and trade are highly varied across the globe, with some more mature in their experiences and thus flexible enough to accommodate the entry of gene edited products for authorization. Although concerns regarding GM crops remain valid and strict legislation requires rigorous scientific assessments in keeping with societal values, too onerous approaches negate the development of scientific expertise and knowledge sharing.

## Author Contributions

CT wrote the manuscript. ML and TH-E provided substantial additions and revised the manuscript. All authors read and approved the final manuscript.

## Conflict of Interest

The authors declare that the research was conducted in the absence of any commercial or financial relationships that could be construed as a potential conflict of interest.

## Footnotes

1 Secretariat of the Convention on Biological Diversity (2000). Cartagena Protocol on Biosafety to the Convention on Biological Diversity: text and annexes. Montreal: Secretariat of the Convention on Biological Diversity. Available at https://bch.cbd.int/protocol/text/ (accessed October 20, 2020).

2 Article 3(g) of the Cartagena Protocol on Biosafety. The Protocol refers to a “living modified organism” in the same way as the ISAAA and many governmental documents usually refer to a “genetically modified organism.”

3 Article 3(i) of the Cartagena Protocol on Biosafety.

4 Paragraph 16 of the Preamble to the Regulation (EC) No 1829/2003.

5 Article 1 of Regulation 1829/2003.

6 Regulation (EC) No 1830/2003 on the traceability and labeling of genetically modified organisms (GMOs) and the traceability of food and feed products produced from GMOs.

7 Article 2(8) of Directive 2001/18/EC.

8 Article 23(1) of Directive 2001/18/EC.

9 Exempted techniques are listed in Annex I A Part 2 and Annex I B of Directive 2001/18/EC.

10 Council Decision (EU) 2019/1904 of 8 November 2019 requesting the Commission to submit a study in light of the Court of Justice’s judgment in Case C-528/16 regarding the status of novel genomic techniques under Union law, and a proposal, if appropriate in view of the outcomes of the study.

11 §1 and §10 of the Gene Technology Act.

12 Unofficial English translation provided by USDA Foreign Agricultural Service (2020c).

13 See the Introduction to the Coordinated Framework for Regulation of Biotechnology.

14 Declaration III (XXXIV 2017) can be found in Spanish at https://tinyurl.com/yybf3kmx (accessed October 13, 2020).

15 Article 401 of the Constitution of the Republic of Ecuador of 2008. English translation can be found at https://tinyurl.com/y5w2d4b5 (accessed October 13, 2020).

16 van Rooij (2012) broadly defines the “bottom-up” development of the law as a mechanism that brings about legal reform and interventions that will improve access to justice or the empowerment of the poor.

17 When it comes to GM crops, MOARA implements the primary regulation (see Table 5) and three supplementary rules related to safety evaluation, import, and labeling measures (Chen and Dai, 2020).
